# Life on the edge: mineral incrustations colonized by fungal communities in the sulfur fumarole on Sierra Negra volcano (Galápagos Archipelago)

**DOI:** 10.1098/rsos.250010

**Published:** 2025-03-05

**Authors:** Maria Landolfi, Raphael Tiziani, Sahra Riviere, Fabio Trevisan, Mariagioia Petraretti, Heinke Jäger, Stefano Cesco, Martin H. Gerzabek, Katharina Keiblinger, Franz Zehetner, Federica Villa, Tanja Mimmo, Luigimaria Borruso

**Affiliations:** ^1^ Faculty of Agricultural, Environmental and Food Sciences, Free University of Bozen-Bolzano, Bolzano, Italy; ^2^ Department of Food, Environmental and Nutritional Sciences, University of Milan, Milano, Italy; ^3^ Charles Darwin Research Station, Charles Darwin Foundation, Santa Cruz, Galapagos, Ecuador; ^4^ Institute of Soil Research, Department of Ecosystem Management, Climate and Biodiversity, BOKU University, Vienna, Austria; ^5^ Competence Centre for Plant Health, Free University of Bozen-Bolzano, Bolzano, Italy

**Keywords:** fungal diversity, extreme environment, fumarole, gypsum, mineralogy

## Abstract

Despite the extensive studies on plant and animal endemism in the Galápagos Islands, fungal diversity remains largely unexplored, particularly in fumarole environments. Here, we explore the fungal diversity in two gypsum incrustations within an active fumarole of Sierra Negra volcano (Isabela Island). We hypothesize that minor differences in the chemical and mineralogical characteristics of these substrates, despite similar environmental conditions, lead to distinct fungal communities with substrate-specialized taxa. Alpha diversity indices showed no significant differences, but beta diversity analysis revealed two distinct fungal communities (PERMANOVA *p* < 0.01), with only 3.31% of operational taxonomic units (OTUs) shared between incrustations and 37.75 and 14.57% uniquely associated with each incrustation. A strong correlation was found between beta diversity and most measured chemical parameters (Mg, S, Fe, Na, Al, Mn, Zn, K, P, Cu). Our findings indicate that even minor differences in the mineral and chemical composition of closely located incrustations significantly influence fungal communities, emphasizing these deterministic factors as key drivers in shaping fungal diversity.

## 1. Introduction

**Figure 1.**
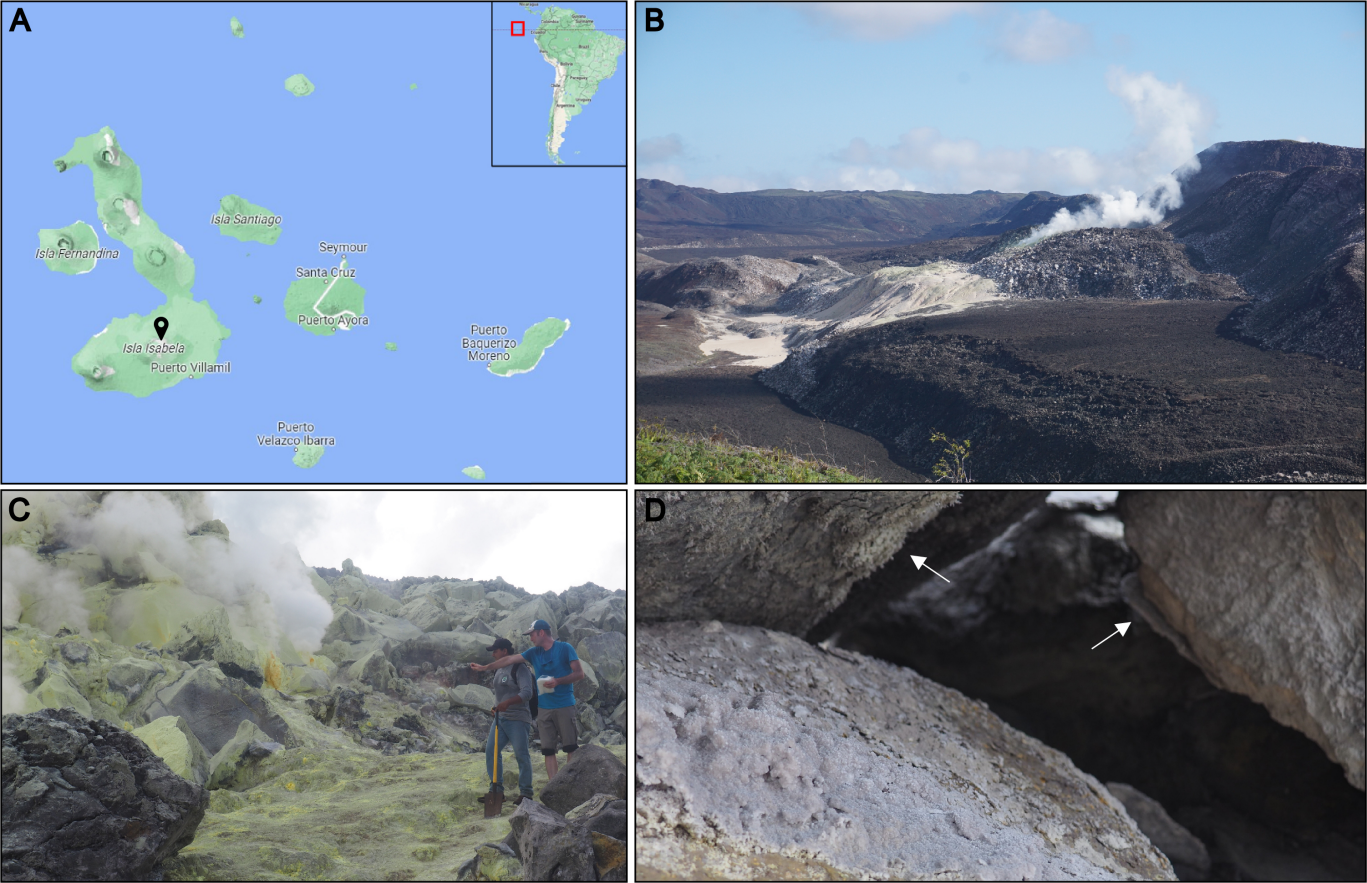
Sampling locations within the Galápagos Archipelago. (A) Map of the Galápagos Archipelago. Black pin marks the location of the Sierra Negra volcano. (B) Azufre fumarole, located in the southwest rim of Sierra Negra caldera (0°49.8159′ S; 91°9.8568567′ W). (C) Detail of the active Azufre fumarole, emphasizing the extreme and arid conditions surrounding the altered basaltic rocks. (D) Detail of the cave, where the two mineral incrustations were collected.

The Galápagos Archipelago, located in the Pacific Ocean at about 1000 km from continental Ecuador, is composed of 15 larger volcanic islands along with numerous smaller islets and occupies a total area of approximately 8000 km^2^ [1] (figure 1A). The Galápagos Islands were formed through volcanic activity caused by a hotspot under the Nazca plate, which is situated near the archipelago’s western boundary. The slow eastward movement of the Nazca plate (55 mm per year) relative to the geostationary hotspot resulted in a chronological pattern where the Galápagos Islands, from west to east, show a progressive increase in age from about 30 000 years to over 1−2 million years [1,2]. Among the volcanoes within the archipelago, Sierra Negra, located on Isabela Island, is one of the most volcanically active and hosts the most intense fumarolic activity of the whole hotspot. The largest fumarolic area here, characterized by flows and encrustations of elemental sulfur (S), is Azufre (figure 1B,C) [3]. Here, elevated temperatures, high S concentrations, limited water and organic matter form an extreme environment around the fumarole and the adjacent altered basaltic rocks.

Although there is no unanimous definition, extreme environments are generally characterized by physicochemical parameters, such as pH, temperature and salinity, significantly differing from values considered ‘normal’ for human life [4–6]. However, a conspicuous diversity of microbial life has been found in extreme environments worldwide [6]. Due to their phenotypic plasticity, extremophilic microorganisms have evolved different adaptive strategies to cope with challenging environmental conditions [7]. These conditions result in lower microbial diversity and simpler communities compared to other environments [8]. The fundamental microbial assemblages, combined with self-sustainability and strong environmental gradients, make these ecosystems a promising model for studying microbial ecology, evolution relationships and analogues of astrobiological investigations [4,9,10]. The research on life in extreme environments has primarily focused on prokaryotes. However, more recent studies have found many eukaryotes, particularly fungi, to be highly adapted to surviving under various stress conditions [11–13].

Fungi are considered the most extreme-tolerant and resilient organisms among extremophiles, displaying remarkable adaptability in both ecological and morphological aspects, along with the ability to thrive with minimal nutrient resources [4,14–16]. Fungi are often the dominant organisms on the oligotrophic surfaces of rocks and minerals, playing a crucial role in mineral transformation and cycling of elements [15]. The geochemical transformations performed by fungi can influence the availability of nutrients for other organisms, facilitating their growth in challenging environments [15], and thus representing an essential resource for these ecosystems. Nevertheless, determining whether a fungal species inhabits the substrate stably or transiently remains unresolved. Indeed, several studies showed that the structure of a microbial community is the result of the complex balance between stochastic and niche-based (or ‘deterministic’) processes [17–19].

Here, we investigated for the first time, to our knowledge, the fungal community associated with two close (approx. 30 cm apart) coloured mineral incrustations collected in a cave located in Minas de Azufre (figure 1D), within the caldera of Sierra Negra volcano (Galápagos Archipelago). We hypothesize that, although the incrustations are exposed to the same macro-environmental conditions, even slight differences in chemical and mineralogical characteristics (i.e. deterministic factors) between the two substrates can result in the selection of distinct fungal communities with substrate-specialized taxa. The rationale for studying mineral incrustations in extreme environments is their relatively low taxonomic complexity compared to other ecosystems, such as soil or sediments [8,20]. This relatively low taxonomic complexity can help to better predict the factors responsible for fungal colonization in these extreme environments.

## 2. Material and methods

### 2.1. Study site and sampling design

Two different coloured mineral materials were collected in a cave near the Azufre fumarole (0°49.8159′ S; 91°9.8567′ W), in the SW rim of the Sierra Negra caldera (Isabela Island, Galápagos, Ecuador; figure 1). The materials were collected using a sterile spatula and gloves and placed in sterile plastic tubes. Four independent replicates from each mineral incrustation type were collected from four distinct spots across their surfaces. Each replicate was subsequently divided into two subsamples: one for the DNA extractions and one for microscopy, mineralogical and chemical analyses. As a result, four replicates for the chemical analyses and four replicates for DNA extraction for each mineral encrustation were obtained. Samples for the DNA analysis were preserved in LifeGuard™ Soil Preservation Solution (QIAGEN, Hilden, Germany) and then stored at −20°C, while samples for chemical analyses were stored in sterile plastic bags at −20°C.

### 2.2. Chemical analyses

For all chemical analyses, samples were homogenized in a Mixer Mill MM 400 (Retsch, Germany). Total carbon (TC) and total nitrogen (TN) were analysed via a Flash EA 1112 elemental analyser (Thermo Fisher Scientific, Waltham, MA, USA). The colour of the minerals was determined using a Chroma Meter CR-400/410 colorimeter (Konica Minolta, Tokyo, Japan), and the difference of colour ΔE76 (δ*E*) was determined using the following equation (Commission Internationale de l’Eclairage (CIE); Colorimetry-technical report, 1986), where L*, a* and b* represent the coordinates in the CIELAB colour space:


(1.1) $$\delta E\;=\sqrt{\delta {\left(L^{*}\right)}^{2}\;+\delta \left(a^{*}\right){\;}^{2}+\delta {\left(b^{*}\right)}^{2}}$$

The pH was analysed by a pH meter (XS Instruments, Carpi, Italy) after an extraction with ultrapure water at a ratio of 1 : 2.5, and centrifugation for 2 min at 1000 r.p.m.

For the elemental analysis, approximately 0.1 g of each sample was acid digested with concentrated ultrapure HNO_3_ (69% w/v) using a single reaction chamber microwave digestion system (UltraWAVE, Milestone, Shelton, CT, USA). After the digestion, ultrapure water was added up to 20 ml, and the solution was filtered at 0.45 µm with cellulose Whatman filter paper (grade 42). Element concentrations were then determined by Inductively Coupled Plasma Mass Spectroscopy (ICP-MS, Agilent 7800, USA), using lanthanum (La) as an internal standard at 1 ppm and normalized by the weight of the sample.

### 2.3. Mineralogical analysis

The bulk mineralogy of the samples was analysed by random powder X-ray diffraction (XRD). The samples were scanned from 2° to 70° 2θ with a step size of 0.017° 2θ and a measuring time of 100 s per step using a PANalytical X’Pert Pro MPD diffractometer equipped with automatic divergent slit, Cu LLF tube (45 kV, 40 mA) and an X’Celerator detector. Mineral identification was conducted with the PANalytical X’Pert HighScore Plus software.

### 2.4. DNA extraction

LifeGuard™ Soil Preservation Solution (QIAGEN, Hilden, Germany) was removed, centrifuging the samples at 2500*g* for 5 min, according to the manufacturer's instructions. Next, approximately 300 mg of each sample were ground with a sterile mortar. Total DNA was then extracted using DNeasy PowerSoil Pro Kits (QIAGEN, Hilden, Germany) with a modification. The mechanical cell lysis was carried out in a Mixer Mill MM 400 (Retsch, Germany), equipped with adapters for the PowerBead Pro Tube provided in the kit. Three cycles, each of 30 s at 30 Hz, were executed, with a 30 s interval between each cycle to prevent excessive sample heating. Since the DNA yield was extremely low (ranging from 0.052 to 0.282 ng/μl), as expected, DNA was concentrated by about threefold in a Savant™ SpeedVac™ SPD120 (Thermo Fisher Scientific, Waltham, MA, USA) to achieve a suitable concentration for library preparation. The final concentration was assessed by a Qubit dsDNA HS Assay (Thermo Fisher Scientific, Waltham, MA, USA) quantification.

### 2.5. Libraries preparation and sequencing

The ITS2 region was amplified by polymerase chain reaction (PCR) using the forward primer ITS3 (5′ GCATCGATGAAGAACGCAGC 3′) [21] and the reverse ITS4 (5′ TCCTCCGCTTATTGATATGC 3′) [21], including the Illumina sequencing adapters attached to their 5′ ends. The PCR was carried out in a final volume of 25 μl, containing 5 μl of the concentrated DNA, 0.6 μM of forward and reverse primers, 12.5 μl of 2× Phusion Flash PCR master mix (Thermo Fisher Scientific, Waltham, MA, USA) and diethyl-pyrocarbonate (DEPC)-treated water up to 25 μl. The reaction mixture was incubated in a BIORAD thermocycler as follows: initial denaturation at 95°C for 5 min, 30 cycles of 95°C for 30 s, 55°C for 30 s, 72°C for 30 s, followed by a final elongation at 72°C for 5 min. The same PCR was carried out on three negative controls: (i) LifeGuard™ Soil Preservation Solution (QIAGEN, Hilden, Germany) used for the storage; (ii) a negative control of DNA extraction; (iii) LifeGuard™ Soil Preservation Solution (QIAGEN, Hilden, Germany) from a new stock. Before proceeding to library indexing, PCR products were purified using the Mag-Bind RXNPure Plus magnetic beads (Omega Biotek, Norcross, GA, USA), following the instructions provided by the manufacturer.

The oligonucleotide indices that are required for multiplexing different libraries in the same sequencing pool were attached by PCR, using universal primers that anneal at the ends of the pre-amplified molecules and that have an oligonucleotide tail containing the corresponding indexes and Illumina-compatible adapters. These PCRs were carried out in a final volume of 25 μl, containing 2.5 μl of the purified PCR products, 1 μM of the dual-indexed primers, 6.5 μl of Supreme NZYTaq 2× Green Master Mix (NZYTech, Lisbon, Portugal), and DEPC water up to 25 μl. The reaction mixture was incubated as follows: an initial denaturation at 95°C for 5 min, followed by 5 cycles of 95°C for 30 s, 60°C for 45 s, 72°C for 45 s, and a final extension step at 72°C for 7 min. A negative control containing no DNA was included to check for contamination during library preparation, and the negative controls were cited above. The library size was checked by running the libraries on 2% agarose gels stained with GreenSafe (NZYTech) and imaging them under UV light. Then, the libraries were purified using the Mag-Bind RXNPure Plus magnetic beads (Omega Biotek), following the instructions provided by the manufacturer. Finished libraries were pooled in equimolar amounts using the Qubit dsDNA HS Assay (Thermo Fisher Scientific, Waltham, MA, USA) quantification. The pool was sequenced with NovaSeq PE250 flowcell (Illumina Inc., San Diego, CA, USA), aiming for a total output of 2 gigabases. The samples have been submitted under accession number PRJNA1138081 in the Sequence Read Archive.

### 2.6. Bioinformatic analysis

The quality of raw data was checked by FastQC [22], and eventual adapter dimers were removed using Cutadapt v. 3.5 [23]. Sequences were pre-processed, quality filtered, trimmed, de-noised, merged, modelled and analysed by DADA2 within QIIME2 [24]. Chimeras were removed using the ‘consensus method’ [25], and subsequently, the sequence variants were grouped via VSEARCH into OTU, utilizing a 97% cutoff [26]. Representative OTUs were taxonomically assigned using the UNITE + INSD database (updated: 22 April 2024) [27] (electronic supplementary material, 1).

All the OTUs found in the four negative controls mentioned in the previous paragraph were classified as ‘contaminants’ and removed from all eight samples to prevent potential external contamination.

### 2.7. Statistical analysis

Statistical analysis of the chemical parameters was performed using either a *t*‐test (*p* < 0.05) [28] or the non-parametric Mann–Whitney *U* test (*p* < 0.05) [29] in cases where data did not follow the assumption of normality. The package ggplot2 in R environment was used for data visualization [30].

Regarding the fungal diversity analysis, the homogeneity in community variance was analysed using PERMDISP [31]. Non-metric multidimensional scaling (NMDS) analysis based on Bray–Curtis distances with the function envfit, of the package ‘vegan’ [32], was performed to highlight the correlation between the chemical parameters and the composition of the fungal communities. The statistical significance of the clustering pattern visualized via NMDS was tested using permutational ANOVA (PERMANOVA) [33]. Relevant fungal OTUs that were significantly associated with each mineral substrate were identified using multivariate analysis by linear models (Maaslin2) in the R environment [34]. Generalists and specialist OTUs for each substrate were determined by a multinomial species classification method using the ‘vegan’ package and the function clamtest in R [35,36]. Principal component analysis (PCA) was performed to analyse the distribution of mineral samples considering their chemical parameters, using prcomp command in R [37].

### 2.8. Imaging by confocal laser scanning microscopy

To visualize fungi and traces of organic materials, mineral samples were stained with Fluorescent Brightener 28 (Sigma-Aldrich, Italy) targeting chitin, and Concanavalin A-Texas red (Invitrogen, Italy) targeting glycoconjugates. The samples were incubated with a staining solution containing 0.1% of Fluorescent Brightener 28 and 100 μg/ml of ConA in phosphate-buffered saline (PBS, 50 mM potassium phosphate, 150 mM NaCl; pH 7.2) for 30 min at room temperature. After the incubation, samples were rinsed five times with warm PBS to remove any unbound dyes.

Images were captured using a confocal laser scanning microscope (Nikon A1R) equipped with a 10× dry lens objective at the UNITECH NOLIMITS Imaging Facility of the University of Milan. Images were acquired using the following excitation laser lines and emission parameters: for Fluorescent Brightener 28 ex 355 nm laser and em 433 nm and for ConA ex 561 nm laser, em 570–620 nm. In addition, the reflection mode was used with an excitation wavelength of 488 nm and an emission wavelength between 480 and 490 nm, enabling the recording of reflective signals originating from the minerals. The obtained images were analysed with the software Imaris Viewer (v. 9.9, Oxford Instruments). Representative images are shown.

## 3. Results and discussion

While the Galápagos Islands have been extensively used as a natural laboratory to study the endemism of various plant and animal species, the diversity of fungi remains largely unexplored [38–40], especially in fumarole environments. Therefore, our study offers a unique opportunity to explore fungal diversity in these extreme environments. The unique samples allowed us to test the hypothesis regarding fungal adaptive strategies and the possible deterministic aspects behind the colonization in gypsum incrustations located in extreme environments.

**Figure 2.**
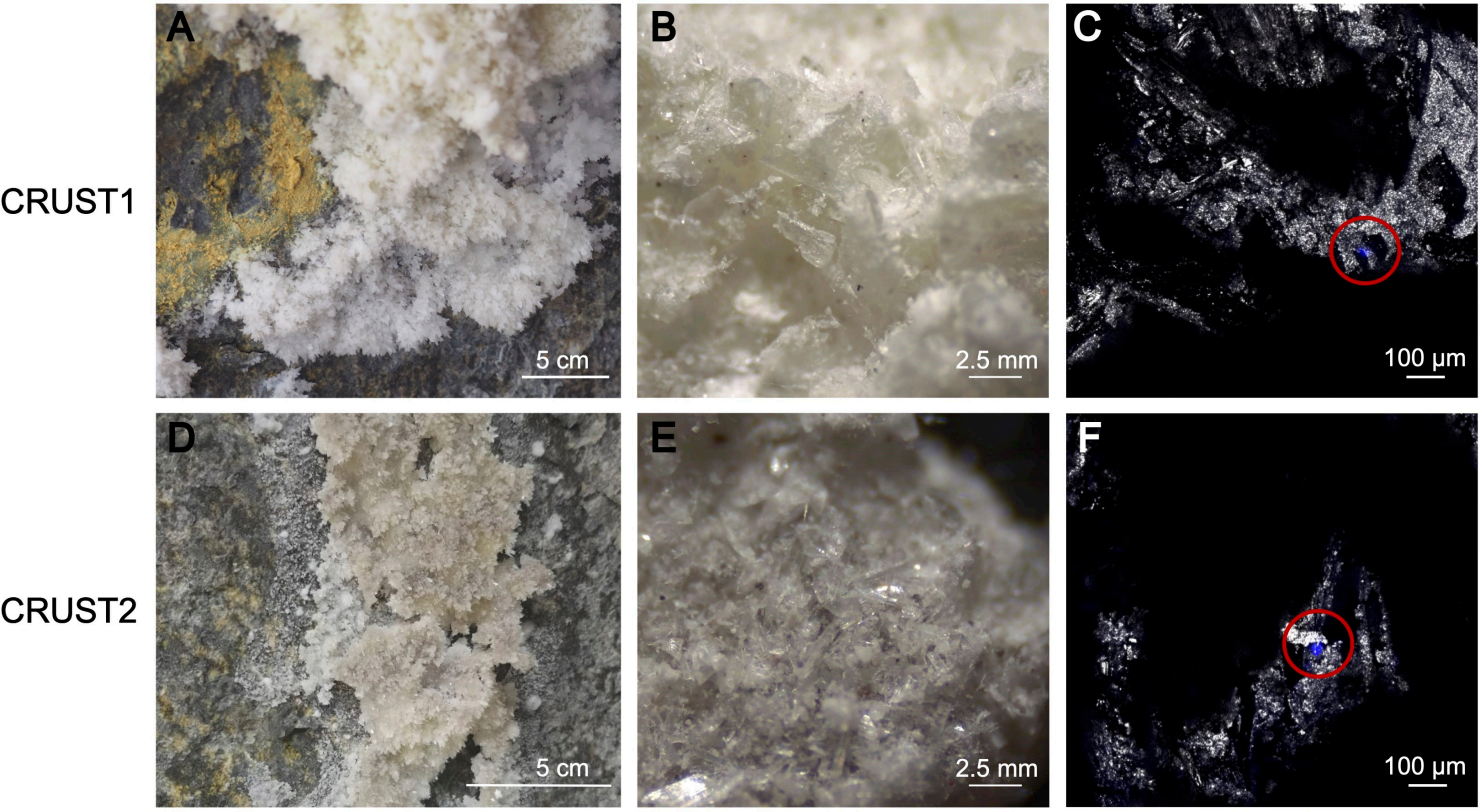
Images showing mineral incrustations collected at different magnifications. Surface view of CRUST1 (A) and of CRUST2 (D), illustrating the distribution of incrustations on the rock surfaces where they were collected. Medium magnification (×20, Leica S9i stereomicroscope) view of CRUST1 (B) and CRUST2 (E), highlighting the granular structure of the crystals. Confocal microscope image of CRUST1 (C) and CRUST2 (F), red circles indicate suspected fungal cells detected using Fluorescent Brightener 28 dye.

**Figure 3.**
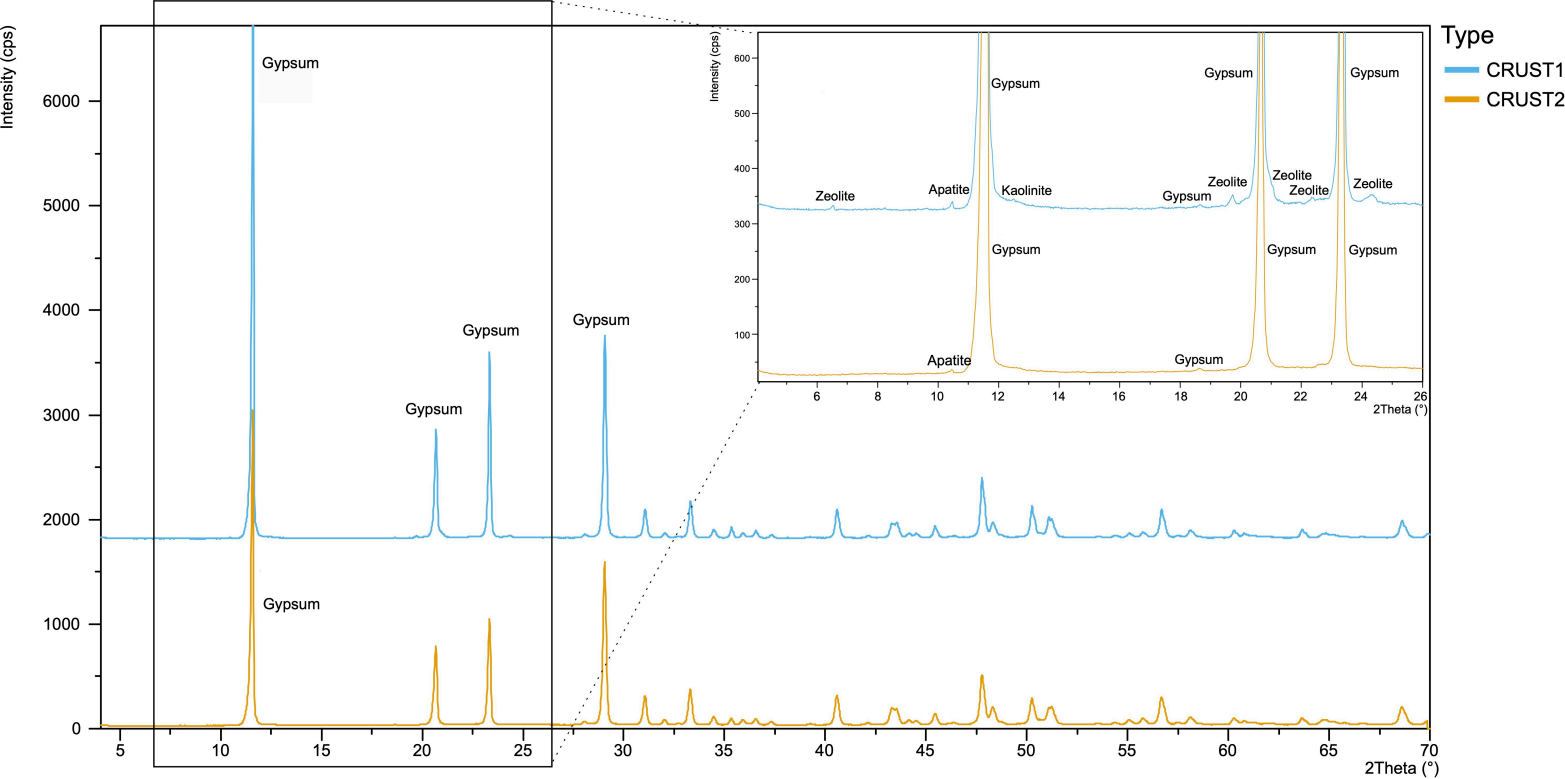
X-ray diffraction (XRD) patterns of the samples from the two mineral incrustations. The inset on the right shows the detail of the diffractogram in the range from 5° to 26°, highlighting the peaks of the minor mineral components.

The brighter white colour with a porous texture of CRUST1 and the grey colour with a grainy texture of CRUST2, observed visually, were confirmed by colorimetric analysis (electronic supplementary material, table S1). The analysis showed a colour difference of δ*E* = ±4.86 (figure 2, electronic supplementary material, figure S1), which, according to the ΔE76 guide, is considered ‘clearly noticeable’ [41]. The mineral incrustations were primarily (>95%) composed of gypsum (CaSO₄•2H₂O) with a few additional minor constituents. Specifically, CRUST1 contained traces of zeolite, apatite and kaolinite, while CRUST2 contained traces of apatite (figure 3).

Gypsum is commonly found in hyperacid volcanic environments rich in sulfates [42–44]. Its porous structure can create microenvironments that trap moisture (particularly during the wet season), potentially providing habitats for microorganisms to survive [45] and offering thermal regulation, structural support and protection against UV radiation [46].

Up to 20.8% of gypsum’s weight can result from water crystallization, being an integral part of the crystal structure of gypsum [47]. The release of this crystallization water can be triggered by weathering of the mineral through environmental factors, such as moisture and temperature changes [47]. Therefore, gypsum can provide water and a protective shelter for microorganisms in arid ecosystems [48,49]. It has also been hypothesized that gypsum may have served as a possible water source for potential past life on Mars [50–52].

Phosphorus (P)-containing mineral apatite (Ca_5_[(F,Cl,OH)|(PO_4_)_3_]) was also present in both samples, albeit in lower percentages, although total P content was fivefold higher in CRUST1 (10.50 µg g^−1^) compared to CRUST2 (1.54 µg g^−1^) (table 1). Fungal species can secrete organic acids (e.g. citrate, malate, oxalate) to release phosphate from apatite, making it a source of the essential macronutrient P [53], thus supporting life in extreme environments [54,55]. In addition, traces of zeolite (Al_2_Si_2_O_5_(OH)_4_) were only detected in CRUST1 (figure 3). Zeolites are primary aluminosilicates with a very high specific surface area due to their porous nature and a high cation exchange capacity. Thus, they represent a potential source of mineral nutrients like calcium (Ca^2+^), magnesium (Mg^2+^), sodium (Na^+^) and potassium (K^+^). In fact, CRUST1 is characterized by significantly higher concentrations of cations compared to CRUST2 (table 1, figure 3). This is further emphasized by the significantly fourfold higher concentration of aluminium (Al), a key structural component of zeolite, detected in CRUST1 compared to CRUST2 (table 1). Additionally, Na, Mg, K and Cu contents were approximately three times higher in CRUST1 than in CRUST2 (table 1). Likewise, the micronutrients Fe, Zn and Mn were present in higher concentrations in CRUST1 than in CRUST2 (table 1). In detail, Mn displayed a significantly 66% higher content, while Fe and Zn showed an approximately twofold concentration in CRUST1 compared to CRUST2 (table 1). Calcium content was similar in both incrustations (around 26 mg g^−1^).

**Table 1.** Chemical parameters of the two mineral incrustations (CRUST1 and CRUST2). The mean concentrations, standard deviation and *p*-values are reported. Means have been compared with a *t*‐test (*p* < 0.05) or the Mann–Whitney *U* test (*p* < 0.05) when data did not respect the assumption of normality. Significantly different *p*-values are in bold, whereas not significant values are abbreviated with n.s. LOD, limit of detection; TC, total carbon; TN, total nitrogen.

chemical parameter	CRUST1	CRUST2	*p*‐value
pH	3.40 ± 0.07	3.39 ± 0.04	n.s.
TC	<LOD	<LOD	—
TN	<LOD	<LOD	—
P (µg g^−1^)	10.50 ± 1.91	1.54 ± 0.31	**0.026**
K (mg g^−1^)	0.08 ± 0.02	0.03 ± 0.01	**0.013**
S (mg g^−1^)	25.45 ± 2.51	18.40 ± 3.52	**0.020**
Ca (mg g^−1^)	26.51 ± 2.14	25.80 ± 3.78	n.s.
Mg (mg g^−1^)	0.57 ± 0.07	0.17 ± 0.03	**0.029**
Na (mg g^−1^)	1.18 ± 0.10	0.40 ± 0.08	**0.029**
Fe (mg g^−1^)	0.55 ± 0.01	0.28 ± 0.03	**0.030**
Zn (µg g^−1^)	6.00 ± 2.82	2.75 ± 0.96	n.s
Mn (µg g^−1^)	28.00 ± 0.81	15.00 ± 1.41	**0.028**
Cu (µg g^−1^)	2.75 ± 1.25	1.25 ± 0.50	**0.044**
Al (mg g^−1^)	1.58 ± 0.17	0.40 ± 0.06	**0.029**

**Figure 4.**
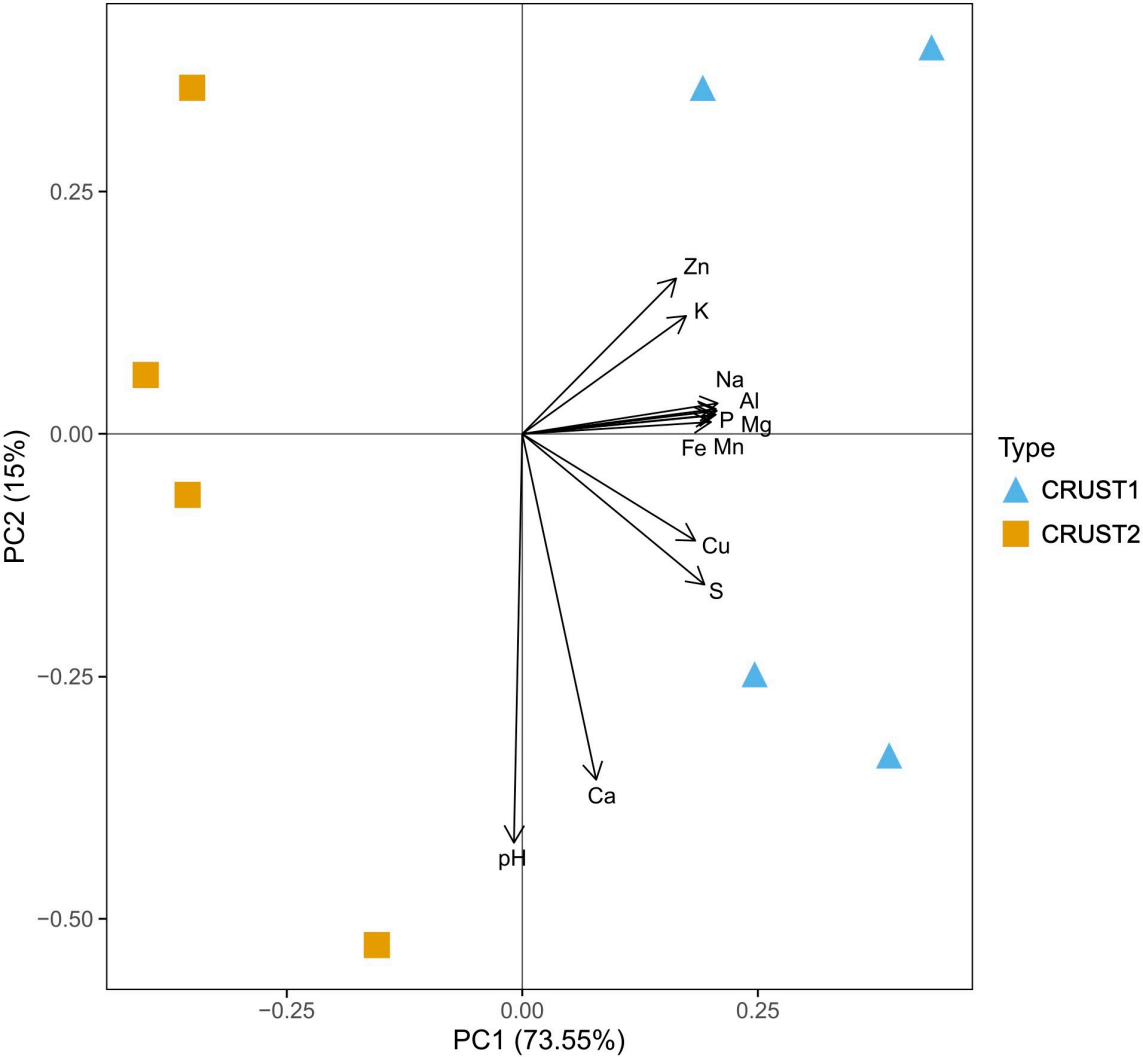
Principal component analysis (PCA) of the two mineral incrustations (CRUST1 and CRUST2), based on pH and elemental composition.

The differences in the chemical composition reflect the different mineralogy of the samples and are underscored by the PCA (figure 4). The PCA effectively separated them into two significantly distinct clusters, with the first and second components accounting for 15.00% and 73.55% of the variation, respectively. The high levels of S (25.45 mg g^−1^ in CRUST1 and 18.40 mg g^−1^ in CRUST2, table 1) can be attributed to the predominance of gypsum as well as emissions of sulfur dioxide (SO_₂_) and hydrogen sulfide (H_₂_S) from the Azufre fumarole and the Sierra Negra caldera [56–58]. These gases are also likely responsible for the acidic pH values observed in the incrustations (3.39 in CRUST1 and 3.40 in CRUST2, table 1) [59]. Both TC and TN were below the detection limit in both mineral incrustations (table 1). This is expected in minerals composed mainly of gypsum and typical of arid environments [60]. Nevertheless, with confocal laser scanning microscopy imaging, we detected minor traces of organic matter (electronic supplementary material, figure S2), which could potentially serve as a source of carbon [61,62].

**Figure 5.**
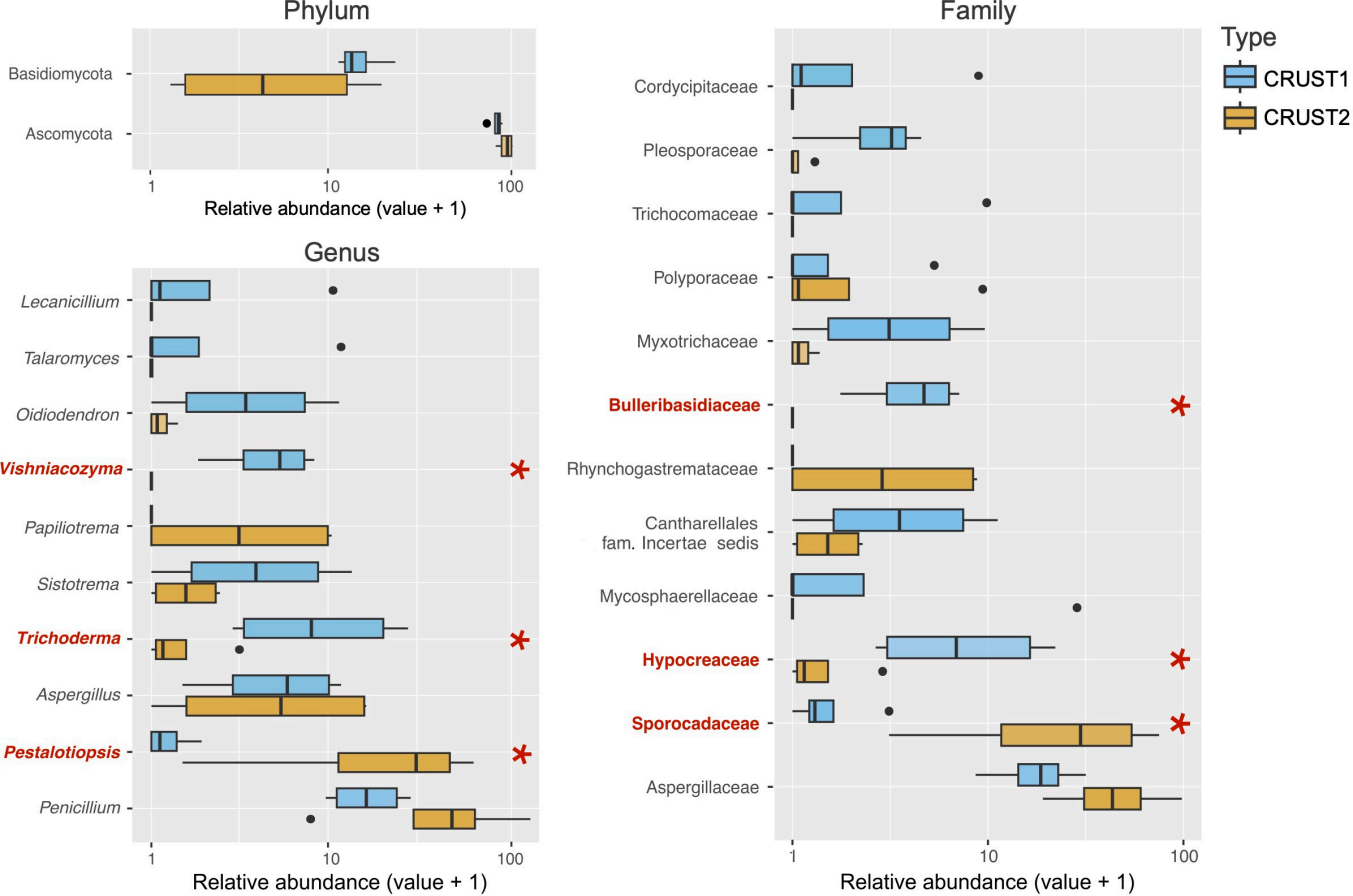
Boxplot showing the relative abundances of fungal OTUs at phylum, family and genus levels. Relevant fungal taxa significantly associated with each mineral substrate identified with Maaslin2 are indicated in bold and red. Stars (*) indicate significance at False Discovery Rate (FDR) <0.05.

The acidic pH contributes further to defining an oligotrophic and extreme environment [5,46]. However, despite these harsh environmental conditions, we identified a considerable fungal diversity (figure 5). We found 151 OTUs belonging to 4 phyla, 15 classes, 35 orders, 52 families and 42 genera, with 12 unidentified OTUs. We excluded all OTUs found in the negative controls (see section 2.5) to avoid potential contamination from the final dataset. This precaution is crucial for accurately representing the microbial biodiversity within samples from extreme environments characterized by low microbial biomass [63]. Indeed, managing the elevated risk of external contamination is one of the major challenges posed by the analysis of life in extreme environments [63]. Overall, the communities of both mineral incrustations were dominated by Ascomycota, followed by Basidiomycota (figure 5). Analysis of alpha diversity indices, including species richness and the Shannon index, revealed no significant differences between the two communities (electronic supplementary material, figure S3). This suggests that both incrustations provide equally favourable conditions for fungal colonization despite their mineralogical differences.

**Figure 6.**
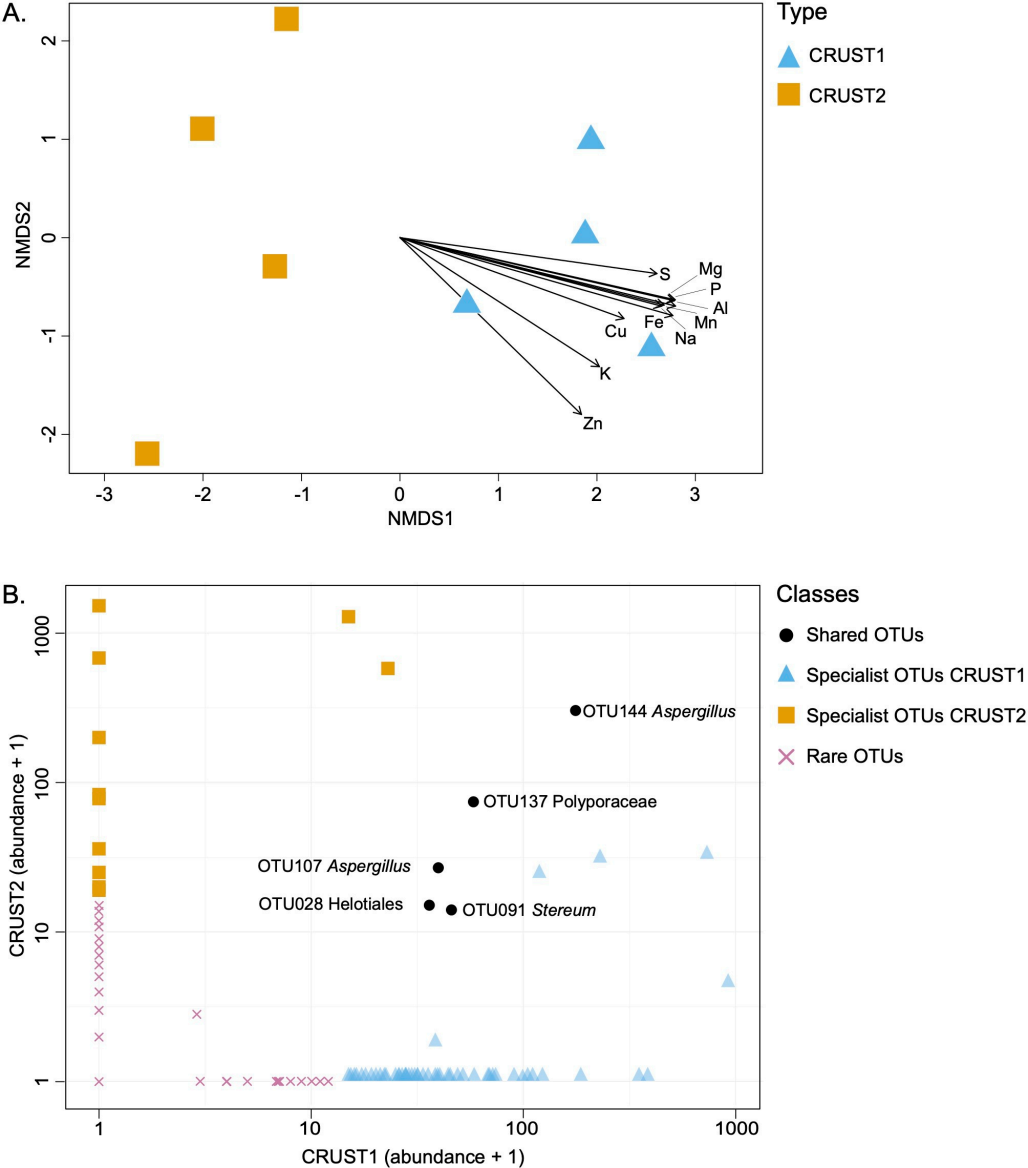
(A) Non-metric multidimensional scaling (NMDS) based on Bray–Curtis distance with envfit function showing the difference between the fungal communities of the two mineral incrustations in correlation with the chemical parameters. (B) Biplot showing the results of the CLAM test based on pairwise comparisons.

However, while the alpha diversity remained unaffected by the chemistry and mineralogy of the incrustations, there was a significant difference in beta diversity of the two fungal communities (figure 6A) (PERMANOVA *p* < 0.01). In support of our hypothesis, the results of the envfit function (electronic supplementary material, table S2) showed that most of the chemical parameters measured in the two mineral incrustations were correlated with the beta diversity, potentially contributing to variations in the fungal assemblage. Nevertheless, both mineral incrustations were inhabited predominantly by rock-associated fungal taxa [64–67]. At the family level, in CRUST1, the abundance of Bulleribasidiaceae with the genus *Vishniacozyma* and Hypocreaceae with *Trichoderma* was higher in CRUST1 than in CRUST2 (figure 5).

These taxa are often found in rocky habitats such as caves and could be crucial in geochemical cycles [62,64,65,67]. Some species affiliated with *Trichoderma* have optimal growth in acidic environments [68] and can produce organic acids, like acetic, citric and formic acids [62], which can solubilize gypsum [69]. Additionally, some *Trichoderma* species can oxidize sulfide, releasing sulfate and aiding in nutrient cycling by enhancing the uptake of Ca²^+^ and SO_₄_²⁻ [62,69]. It has also been demonstrated that some species within the *Vishniacozyma* genus can solubilize P by producing tartaric and acetic acids, enhancing phosphate availability for themselves and other microorganisms [64]. In contrast to CRUST1, CRUST2 had a higher abundance of Sporocadaceae, primarily represented by the genus *Pestalotiopsis*. Some works suggest that species within this genus can facilitate calcite precipitation through urease secretion in certain conditions [15,70]. The significant difference between the fungal communities in our samples is further highlighted when examining specialist and shared OTUs [35]. We observed that 37.75% of OTUs were specialist in CRUST1 and 14.57% in CRUST2 (see figure 6B). In CRUST1, the specialist OTUs were affiliated with Trichocomaceae, Pleosporaceae and Myxotrichaceae. Members of these families have been discovered in caves, rocks and other extreme environments such as the Atacama Desert and high mountain environments [12,20,66,71–73], suggesting their ability to thrive in dry and oligotrophic conditions. Also, in CRUST2, we found OTUs affiliated with *Penicillium*, previously described in different endolithic microhabitats and able to survive in acidic environments [74]. Only five OTUs were generalist, i.e. shared between the mineral incrustations (3.31%). These OTUs belong to the taxa of Helotiales (OTU028), Russulales (OTU091), Polyporales (OTU137) and *Aspergillus* (OTU107 and OTU144), known to have a wide distribution and the ability to thrive in harsh and oligotrophic conditions [46,75–77].

## 4. Conclusion

In conclusion, the analysis of fungal communities, mineral composition and chemical characteristics of the two incrustations in our study confirmed the specificity of fungal communities associated with a particular mineral composition. The presence of a few shared OTUs and a high percentage of specialist taxa again supports the hypothesis that the composition of mineral incrustations plays a crucial role in selecting and shaping the fungal community [78]. Our results suggest that deterministic factors, such as the mineral and chemical compositions of the substrates, could contribute to the differentiation of the fungal communities. Since the CRUST1 and CRUST2 samples are in the same confined environment, exposed to the same macro-environmental conditions and only about 30 cm apart, the characteristics of the mineral incrustations are likely among the main factors shaping the fungal community.

## Data Availability

DNA sequences obtained with the sequencing and used for the analyses have been submitted under accession number PRJNA1138081 in the Sequence Read Archive (SRA). Supplementary material is available online [79].
